# Development of Stable Infectious cDNA Clones of Tomato Black Ring Virus Tagged with Green Fluorescent Protein

**DOI:** 10.3390/v16010125

**Published:** 2024-01-15

**Authors:** Aleksandra Zarzyńska-Nowak, Julia Minicka, Przemysław Wieczorek, Beata Hasiów-Jaroszewska

**Affiliations:** 1 Department of Virology and Bacteriology, Institute of Plant Protection—National Research Institute, Wladyslawa Wegorka 20, 60-318 Poznan, Poland; j.minicka@iorpib.poznan.pl (J.M.); b.hasiow@iorpib.poznan.pl (B.H.-J.); 2 Department of Molecular Biology and Biotechnology, Institute of Plant Protection—National Research Institute, Wladyslawa Wegorka 20, 60-318 Poznan, Poland; p.wieczorek@iorpib.poznan.pl

**Keywords:** infectious cDNA clone construction, GFP, tomato black ring virus, FMDV 2A peptide

## Abstract

Tomato black ring virus (TBRV) is a member of the *Nepovirus* genus in the *Secoviridae* family, which infects a wide range of important crop species worldwide. In this work, we constructed four cDNA infectious clones of the TBRV tagged with the green fluorescent protein (TBRV-GFP), which varied in (i) the length of the sequences flanking the GFP insert, (ii) the position of the GFP insert within the RNA2 polyprotein, and (iii) the addition of a self-cutting 2A protein. The presence of the GFP coding sequence in infected plants was verified by RT-PCR, while the infectivity and stability of the constructs were verified by mechanical inoculation of the host plants. The systemic spread of TBRV-GFP within plants was observed under UV light at a macroscopic level, monitoring GFP-derived fluorescence in leaves, and at a microscopic level using confocal microscopy. The obtained clones are a valuable tool for future studies of TBRV-host interactions, virus biology, and the long-term monitoring of its distribution in infected plants.

## 1. Introduction

Infectious clones of plant viruses are engineered constructs containing a transcriptionally controlled full-length genome copy derived from the wild-type virus. Hence, infectious clones are a source of virus RNA/DNA that is able to replicate, infect, and spread in the host. Since 1984, when the first RNA infectious clone was established (developed for brome mosaic virus, BMV) [1] a variety of plant viruses from different families, such as *Potyviridae* [2,3,4,5], *Virgaviridae* [6,7,8,9], *Luteoviridae* [10,11,12], *Secoviridae* [13,14,15], *Geminiviridae* [16], and many others, have been reconstituted from their infectious clones. Using the constructs, combined with site-directed mutagenesis and an efficient inoculation method (RNA transcripts or agroinoculation), provides numerous opportunities to assay the biological activity and functionality of the studied viruses [17]. The infectious clones are valuable tools for studying various aspects of plant virology, including virus–host interactions, replication mechanisms, or the analysis of plant resistance strategies. Moreover, the incorporation of the visual marker, such as green fluorescent protein (GFP), into the constructs enables studying viral cell-to-cell or vascular movement. Such constructs also serve as a platform for the development of genetically engineered viruses for crop improvement or virus-induced gene silencing studies. Additionally, the infectious clones can be used to produce viral vectors for the expression of foreign genes in plants, making them important tools in plant molecular biology and biotechnology.

Tomato black ring virus (TBRV) is a member of the *Nepovirus* genus (subgroup B) in the family *Secoviridae,* which infects over 90 species, including important crops, berry fruits, ornamental, herbaceous, and woody plants [18]. On infected host plants, the TBRV causes mosaic or necrosis on the leaves, with the most characteristic symptoms of necrotic rings on the leaves and fruits. The virus can be easily transmitted mechanically by nematodes (*Longidorus attenuates*, *L. elongatus*) and through seeds, which promotes the dissemination of the virus over long distances [18,19]. Since its first description in 1946 in the UK, the TBRV has been found in 40 countries [18,20]. There have been many reports of significant damage caused by TBRV infection to several important hosts, e.g., artichokes and grapevines, reaching 40% and 60% of yield losses, respectively [21,22]. The latest reports from Poland have shown the virus presence in 7.6% and 1.2% of tested blackberry and raspberry samples, respectively [23], and 53% of tested tomato samples from Saudi Arabia [24]. Taking into account TBRV properties, host range, distribution, and its infectivity to the propagative host plants, the virus has been subjected to compulsory monitoring and control (regulated pest on wild strawberries, blackberries, gean, and cherry plants listed in Annex IV/Part J of EU Directive 2019/2072) in the European Union.

The genome of TBRV consists of two ssRNA molecules: RNA1 and RNA2, each with VPg on the 5′ and polyA tail on the 3′ end. The genomic RNAs are initially translated into long polyproteins, which are cleaved subsequently into functional proteins. The proteins generated from RNA1 are responsible for virus replication and polyprotein maturation, while RNA2 encodes proteins involved in virion formation and virus movement. In addition, the genomic RNAs of some TBRV isolates might be accompanied by subviral particles such as defective RNAs (D RNAs) and satellite RNAs (satRNAs) [25,26,27]. These particles are relatively short and non-infectious, and their replication, encapsidation, and spread are strictly dependent on the helper virus [28]. It has been shown that TBRV D RNAs might be generated from both genomic RNAs during prolonged passages in one host [27,29,30]. TBRV D RNA structure is diverse, contains parts of non-coding regions, and preserves a portion of the open reading frames of their helper virus genome [27,29,30]. The presence of subviral RNAs may have an impact on viral replication, accumulation, and the expression of symptoms observed in infected plants [27,31,32]. It has been shown that D RNAs interfere with TBRV replication; however, the impact depends on the interplay between TBRV isolates and host species [27]. Satellite RNAs are another type of subviral RNA that can be associated with TBRV genomes. TBRV satRNAs consist of a single-stranded RNA of about 1375 nt that encodes a protein of 48 kDa, which probably affects the activity of the helper virus replicase [31,33,34]. Results described recently showed that the presence of satRNAs significantly impacts TBRV accumulation, depending on the virus isolate and the infected host plant [31].

In our previous studies, we reported the construction of a full-length infectious clone of three TBRV isolates originating from tomato (TBRV-P1) [15], zucchini (TBRV-K8), and black locust (TBRV-M1) [34]. In this paper, we describe different attempts at engineering TBRV-P1 cDNA clones fused with GFP. The four engineered clones varied in (i) the length of the sequences flanking the GFP insert, (ii) the position of the GFP insert within the RNA2 polyprotein, and (iii) the addition of a self-cutting 2A protein sequence facilitating the release of the GFP from the virus polyprotein. The biological activity and stability of the virus constructs were confirmed by their mechanical inoculation into the host plant, while the local and systemic spread of the recombined virus within the infiltrated plants was examined under UV light and confocal microscopy.

## 2. Materials and Methods

### 2.1. Generation of GFP-Tagged Infectious TBRV Clones Utilizing the In-Fusion Cloning Method

**Figure 1:**
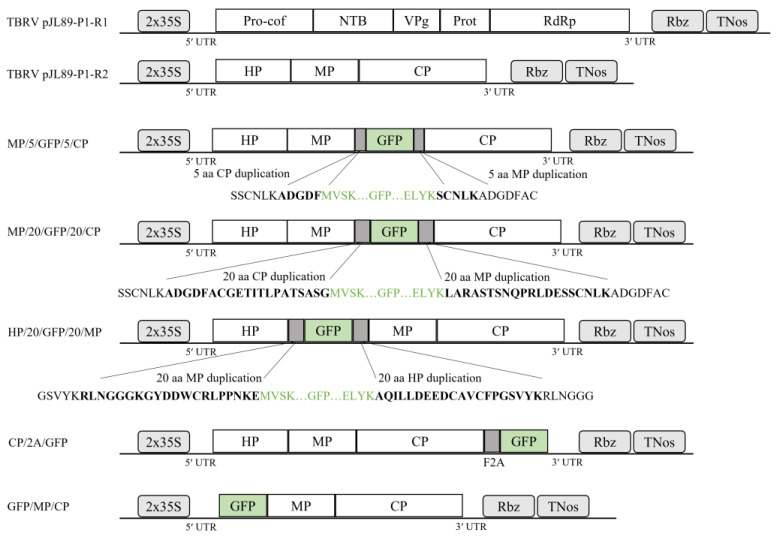
Schematic representation of recombined plasmids used in the study. TBRV-pJL89-P1-R1 and TBRV-pJL89-P1-R2 cDNA infectious clones comprise the full genomes of TBRV RNA1 and TBRV RNA2, respectively. Boxes represent RNA1- and RNA2-encoded polyproteins with their putative functional motifs: Pro-cof: protease cofactor, NTB: nucleotide triphosphate-binding protein, VPg: viral genome-linked protein, Prot: cysteine-like protease, Pol: RNA-dependent RNA polymerase (RdRp), HP: homing protein, MP: movement protein, and CP: capsid protein. The duplicated 35S promoter (2 × 35S) of the cauliflower mosaic virus is located immediately upstream of the first nucleotide of both cDNAs. The hepatitis delta virus ribozyme (Rbz) and the nopaline synthase terminator (TNos) are engineered in tandem after 20 adenines (poly-A tail). The MP/5/GFP/5/CP and MP/20/GFP/20/CP clones comprise the full genome of TBRV-RNA2, and the green fluorescent protein (GFP) coding sequence is inserted between the MP and the CP, flanked by 5 or 20 amino acids, respectively. The HP/20/GFP/20/MP clone consists of the full genome of TBRV-RNA2 with the GFP coding sequence inserted between the HP and the MP, flanked by 20 amino acids. Duplicated putative protease recognition sites between the MP and the CP, and HP and MP, are in bold. The GFP N-terminal and C-terminal amino acid sequences are marked in green. The CP/2A/GFP clone consists of the full genome of TBRV-RNA2 with a GFP-coding sequence fussed with the C-terminus of the foot-and-mouth disease virus (FMDV) 2A self-cleaving peptide.

To engineer a GFP-tagged cDNA TBRV construct, the previously obtained cDNA infectious clones (named pJL89-P1-R1 and pJL89-P1-R2) of the TBRV-P1 isolate were used [15]. A set of primers (Supplementary Table S1) was designed using the SnapGene software 4.0.8 (https://www.snapgene.com, accessed on 8 July 2016) to (1) linearize the plasmid vector and (2) amplify the GFP coding sequence. The pJL89-P1-R2 used as the backbone for the insertion of the EGFP coding sequence in different regions of RNA2 (Figure 1) was prepared by CloneAmp HiFi PCR Premix (Takarabio, Kusatsu, Japan) and appropriative primer pairs (Supplementary Table S1). The GFP coding region was amplified from EGFP-containing plasmids kindly provided by Dr. Massimo Turrina (Institute for Sustainable Plant Protection, CNR-Torino), CloneAmp HiFi PCR Premix (Takarabio, Kusatsu, Japan), and appropriative primer pairs (Supplementary Table S1). All amplicons were gel-purified using the Zymoclean Gel DNA Recovery Kit (Zymo Research, Irvine, CA, USA) according to the manufacturer’s instructions. In-Fusion reaction was performed using an insert-to-vector ratio of 3:1, 5× In-Fusion HD Enzyme Premix (Takarabio, Kusatsu, Japan), and water to 10 µL of total volume. The reaction was incubated at 50 °C for 15 min and then transformed into *Escherichia coli* competent cells (DH5α) (Thermo Fisher Scientific, Waltham, MA, USA). The obtained clones were screened by colony PCR with primer sets overlapping the engineering region of EGFP insertion (Supplementary Table S2 and File S1). The presence of the GFP coding sequence in recombined RNA2 was confirmed by Sanger sequencing (Genomed S.A., Warsaw, Poland). Additionally, in the CP/2A/GFP construct, the coding sequence of the reporter gene was inserted downstream of the 2A self-cleaving peptide (GSGVKQTLNFDLLKLAGDVESNPGP) of the foot-and-mouth disease virus (FMDV) (2A-FMDV). For this, two oligos, 2AFMDV_F/2AFMDV_R (Supplementary Table S3), were mixed 1:1 in water, incubated at 95 °C for 5 min, and gradually cooled at room temperature (RT), allowing oligo self-annealing. After gel electrophoresis, the oligo duplex was extracted from the gel and ligated to the pJET1.2/blunt Cloning Vector (Thermo Fisher Scientific, Waltham, MA, USA), followed by cloning in *E. coli* and Sanger sequencing (Genomed S.A., Warszawa, Poland). The resulting pJet1.2-2A-FMDV plasmid was used as a template in a PCR reaction using CloneAmp HiFi PCR Premix (Takarabio, Kusatsu, Japan). The 2A-FMDV PCR product was purified from agarose and cloned using an In-Fusion HD Cloning kit (Takarabio, Kusatsu, Japan) on the 3′ end of the pJL89-P1-R2 backbone. The resulting pJL89-P1-R2-2A plasmid was used as a backbone for engineering the CP/2A/GFP construct (Figure 1), where the EGFP coding sequence was inserted downstream of the 2A in the pJL89-P1-R2-2A. Primer sets used for engineering this clone are included in Supplementary Table S1. 

### 2.2. Agrobacterium Infiltration 

The binary plasmids (~100 ng/μL) were electroporated into the *Agrobacterium tumefaciens* GV3101 strain as previously described [15]. Bacterial cell suspensions were adjusted to an optical density (OD_600_) of 0.8–1.2 and incubated for 2 to 4 h at room temperature (RT). Before agroinfiltration, equal volumes (1:1:1) of *Agrobacterium* cultures harboring pJL89-P1-R1, an appropriate TBRV pJL89-P1-R2-GFP clone (MP/5/GFP/5CP, MP/20/GFP/20/CP, HP/20/GFP/20/MP, or CP/2A/GFP), and pBIN61-p19 expressing the P19 protein from tomato bushy stunt virus (TBSV), the well-known suppressor of RNA silencing, were mixed. The *Nicotiana tabacum* cv. Xanthi (at the four- to six-leave stage) plants were infiltrated using a 2 mL syringe without a needle. Mock-infiltrated plants were used as negative controls. Plants were grown under greenhouse conditions of 16 h of light and 8 h of darkness at 24/22 °C, respectively. 

### 2.3. TBRV and GFP Coding Sequence Detection 

After 20 dpi from each agroinfiltrated plant, 100 mg of upper non-infiltrated leaves were used for total RNA isolation using the RNeasy Plant Mini Kit (Qiagen, Hilden, Germany) according to the manufacturer’s instructions. The obtained total RNAs were used in RT-PCR performed in a 50 μL mixture containing 25 μL of DreamTaq Green PCR Master Mix (Thermo Fisher Scientific, Waltham, MA, USA), 1 μL of RevertAid Reverse Transcriptase (Thermo Fisher Scientific, Waltham, MA, USA), 2 μL of primers flanking the coding sequence of the GFP insert (Supplementary Table S2 and File S1) and 22 μL of sterile water. The reaction conditions were as follows: reverse transcription at 42 °C for 20 min, 94 °C for 3 min, followed by 30 cycles of 94 °C for 30 s, 55 °C (for each primer pair) for 30 s, 72 °C for 1 min, and a final elongation at 72 °C for 5 min. RT-PCR products were verified by electrophoresis in 1% agarose gel, purified by Zymoclean Gel DNA Recovery Kits (Zymo Research, Irvine, CA, USA), and Sanger-sequenced by an outsourced company (Genomed S.A., Warsaw, Poland).

### 2.4. Confirmation of Biological Activity, Stability, and Infectibility of Constructed Clones

To confirm the biological activity of constructed clones, four host plants: *Cucumis sativus* (at two fully expanded cotyledons and one true leaf stage), *Chenopodium quinoa* (at two cotyledons and four true leaf stages), *Nicotiana tabacum* cv. Xanthi (at four- to six-leaf stages) and *N. benthamiana* (at four- to six-leaf stages) were agroinfiltrated by pJL89-P1-R2-GFP clones. The infectibility and stability were carried out by mechanical transmissions, as previously described [35]. Briefly, approximately 100 mg of *N. tabacum* cv. Xanthi plants were collected 20 days after infiltration (dpi) and grounded in 0.05 M phosphate buffer (pH 7.0). The prepared sap was used to inoculate carborundum-dusted plants of *N. benthamiana* and *N. tabacum* cv. Xanthi, *C. quinoa*, and *C. sativus*. Each time, three plants were used as biological repetitions. 

Twenty days after agroinfiltration/mechanical transmission, virus infection and the presence of the GFP open reading frame were verified in plants by RT-PCR as described above, whereas the UV lamp was used to visualize GFP-derived fluorescence in tested plants.

### 2.5. GFP Expression Observation

The agroinfiltrated and non-infiltrated leaves were assessed for GFP fluorescence at 3 and 7 dpi, or 10 and 20 dpi, respectively, using a UV lamp (Vilber, Lourmat, Germany). Simultaneously, two tissue discs (5 mm in diameter each) were collected from the same plants and prepared for fluorescence microscopy observation. Fluorescence microscopy analysis was performed using a BX53 microscope (Olympus, Tokyo, Japan) with a GFP-specific filter. Laser scanning confocal microscopy observation was performed at the Laboratory of Electron and Confocal Microscopy (Faculty of Biology, Adam Mickiewicz University, Poznań, Poland) using a Zeiss LSM 510 confocal microscope (Zeiss, Jena, Germany). The samples were prepared as described above. Fluorescence was measured using a 488 nm emission wavelength.

## 3. Results

### 3.1. Construction of TBRV pJL89-P1-R2-GFP Infectious Clones

To create stable and infectious clones expressing the GFP open reading frame in infected plants, four varied constructs were engineered. The GFP coding sequence of the first two constructs, named MP/5/GFP/5CP and MP/20/GFP/20/CP, was inserted between movement protein (MP) and coat protein (CP) (Figure 1). The third clone, named HP/20/GFP/20/MP, contained a GFP coding sequence between the homing protein (HP) and MP. The GFP coding sequence was flanked at the N- and C-terminal regions with putative protease recognition sites corresponding to the insertion loci by 5 or 20 amino acids, respectively (Figure 1). In the CP/2A/GFP construct, the coding sequence of the reporter gene was inserted downstream of the 2A self-cleaving peptide of FMDV (Figure 1).

**Figure 2:**
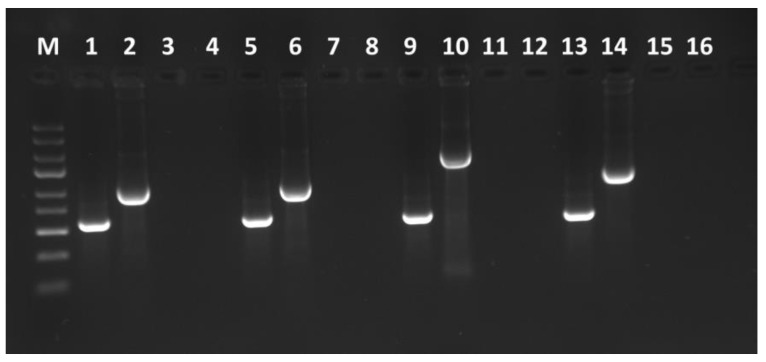
Electrophoretic separation of RT-PCR products from *N. tabacum* cv. Xanthi plants agroinfiltrated with pJL89-TBRV-P1-R1 (1, 5, 9 and 13) and pJL89-TBRV-P1-R2-GFP constructs: MP/5/GFP/5CP (2), MP/20/GFP/20/CP (6), HP/20/GFP/20/MP (10), CP/2A/GFP (14) and mock inoculated plants (3, 7, 11, 15 and 4, 8, 12, 16 for pJL89-TBRV-P1-R1 and pJL89-TBRV-P1-R2-GFP, respectively).

The GFP coding sequence was amplified from the received plasmid and introduced into the TBRV-pJL89-P1-R2 vector via the In-Fusion cloning method. Tobacco is known to support the amplification and foreign gene expression of most established plant viral vectors [36]; therefore, the *N. tabacum* cv. Xanthi was chosen to perform the agroinfiltration and test clone infectibility. At twenty-five dpi, the presence of pJL89-TBRV-P1-R1 and pJL89-TBRV-P1-R2-GFP in infiltrated plants was analyzed by RT-PCR using primers amplifying a fragment of TBRV RNA1 and flanking the engineered region of RNA2 (Supplementary Table S2 and File S1). The RT-PCR products of the expected size of ~600 bp for the RNA1 fragment and 980 bp, 1070 bp, 1950 bp, and 1280 bp for MP/5/GFP/5/CP, MP/20/GFP/20/CP, HP/20/GFP/20/MP, and CP/2A/GFP, respectively, were detected (Figure 2). No RT-PCR products were obtained for mock-inoculated plants.

### 3.2. Biological Activity of TBRV pJL89-P1-R2-GFP Clones in Various Hosts

**Figure 3:**
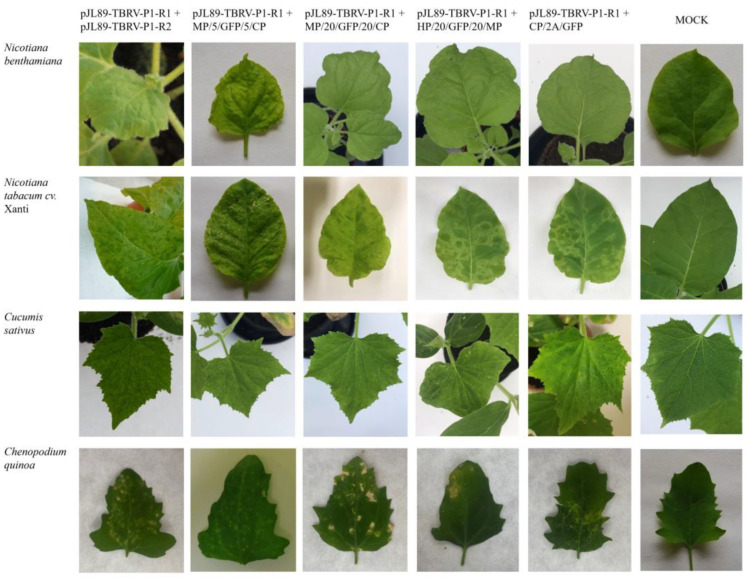
Symptoms of TBRV-GFP were observed on the upper non-infiltrated leaves of *N. benthamiana*, *N. tabacum* cv. Xanthi, *C. sativus*, and *C. quinoa* plants agroinfiltrated with the four tested pJL89-TBRV-P1-R2-GFP clones, namely: pJL89-TBRV-P1-R1 + MP/5/GFP/5CP, pJL89-TBRV-P1-R1 + MP/20/GFP/20/CP, pJL89-TBRV-P1-R1 + HP/20/GFP/20/MP, and pJL89-TBRV-P1-R1 + CP/2A/GFP. The first column shows symptoms on host plants caused by TBRV-P1 derived from its infectious clone (pJL89-TBRV-P1-R1 + pJL89-TBRV-P1-R2), while the last one demonstrates mock-infiltrated plants.

The biological activity of the constructed TBRV-GFP clones was then analyzed using four host plant species: *N. benthamiana*, *N. tabacum* cv. Xanthi, *C. sativus*, and *C. quinoa*. Visible symptoms such as yellow spots, yellow mosaic, and ring mosaic on upper non-infiltrated leaves were observed at 15–20 dpi on *N. tabacum* cv. Xanthi, *C. quinoa*, and *C. sativus* plants for all four constructs (Figure 3). The symptoms developed on *C. quinoa* plants appeared earlier and were more severe compared to other hosts (visible necrotic spots on leaves) and led to plant death at 25–30 dpi. There were no visible symptoms in *N. benthamiana* infected by TBRV-GFP clones (Figure 3).

At twenty-five dpi, the presence of pJL89-TBRV-P1-R1 and pJL89-TBRV-P1-R2-GFP clones in infiltrated tested plants was analyzed by RT-PCR as described above. The infection efficiency of different constructs varied depending on the used constructs and host (Table 1).

### 3.3. Systemic Expression of the Gfp Gene in Tested Plant Hosts

**Figure 4:**
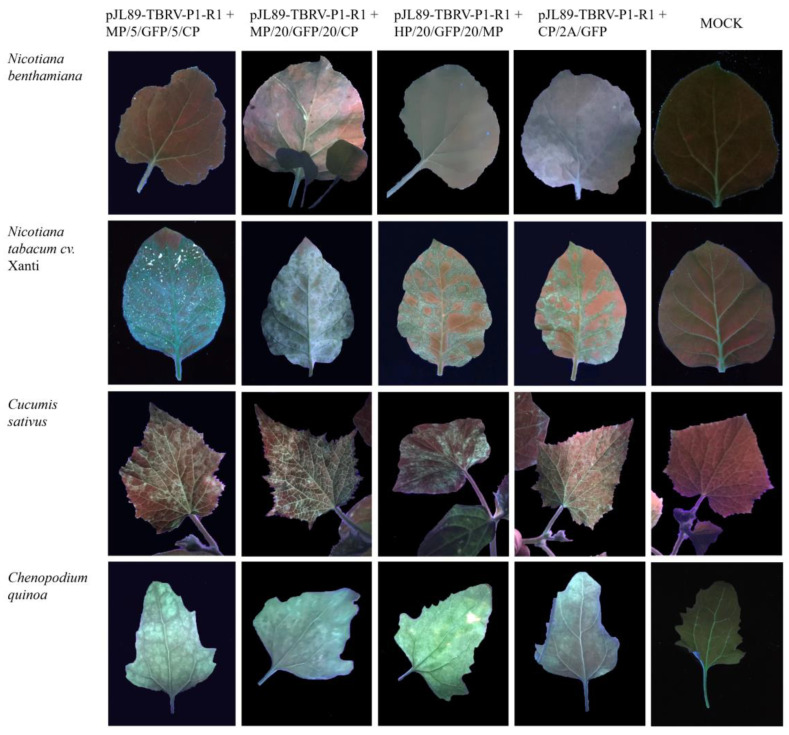
GFP fluorescence under UV light (Vilber, Lourmat, Germany) of upper non-inoculated leaves of *N. benthamiana*, *N. tabacum*, *C. sativus*, and *C. quinoa* agroinfiltrated with four constructed pJL89-TBRV-P1-R2-GFP clones: pJL89-TBRV-P1-R1 + MP/5/GFP/5CP, pJL89-TBRV-P1-R1 + MP/20/GFP/20/CP, pJL89-TBRV-P1-R1 + HP/20/GFP/20/MP, and pJL89-TBRV-P1-R1 + CP/2A/GFP at 20 dpi.

At three and seven dpi, the GFP fluorescence of agroinfiltrated leaves was observed under UV light in all tested plants agroinfiltrated with all constructs. The same was conducted at 20 dpi in the upper non-inoculated leaves (Figure 4). The GFP fluorescence was observed for *N. tabacum* cv. Xanthi, *C. sativus*, and *C. quinoa* plants, while very slight or no signal was noticed for symptomless *N. benthamiana* leaves.

**Figure 5:**
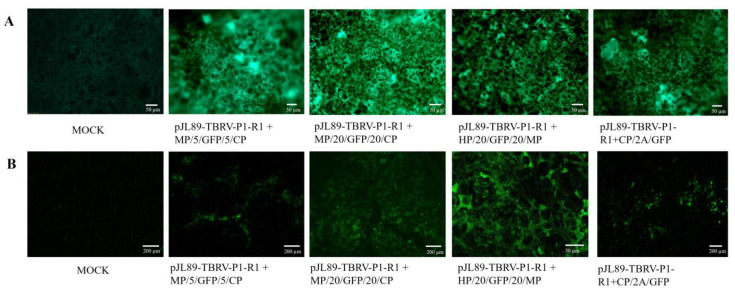
GFP fluorescence observation of upper non-inoculated leaves of *N. tabacum* cv. Xanthi plants infected by pJL89-TBRV-P1-R1 + MP/5/GFP/5CP, pJL89-TBRV-P1-R1 + MP/20/GFP/20/CP, pJL89-TBRV-P1-R1 + HP/20/GFP/20/MP, and pJL89-TBRV-P1-R1 + CP/2A/GFP constructs at 20 dpi using fluoresce BX53 microscope (Olympus, Tokyo, Japan) (**A**) and Carl Zeiss LSM510 confocal microscope (Zeiss, Jena, Germany) (**B**). Mock-inoculated plants were used as a negative control.

The inoculated leaves and upper non-inoculated leaves were also examined by fluorescence and confocal microscopy. For all constructs, GFP fluorescence was detected (Figure 4 and Figure 5). No GFP fluorescence was observed on mock plants (Figure 4 and Figure 5). 

### 3.4. Stability Confirmation of Constructed pJL89-TBRV-P1-R2-GFP Clones

To confirm the stability of pJL89-TBRV-P1-R2-GFP clones, mechanical inoculation of *N. benthamiana*, *N. tabacum* cv. Xanthi, *C. sativus*, and *C. quinoa* was performed (Supplementary File S2). After 15–20 dpi, symptoms such as yellow spots and yellow mosaic were observed on non-inoculated leaves of *N. tabacum* cv. Xanthi, *C. quinoa*, and *C. sativus* plants for all four constructs. *N. benthamiana* plants remain symptomless. The presence of the GFP coding sequence was confirmed by RT-PCR (using the same methodology as described above) (Supplementary File S2). The GFP fluorescence was observed under UV light (Supplementary File S2). These results confirmed the presence of each pJL89-TBRV-P1-R2-GFP clone in infected plants and its stability through mechanical transmission.

## 4. Discussion

Infectious clones of plant viruses are important tools with wide-ranging applications in different areas of biology and medicine. Infectious cDNA clones can be used to study important virus properties such as virulence/attenuation, cell penetration, replication, host range, and functions of coding or non-coding genomic regions. The infectious clones can be applied in plant pathology, including the study of plant–virus interactions and screening of germplasm as part of prebreeding programs for virus resistance. Plant viruses are also increasingly being investigated as expression vectors in planta production of pharmaceutical products [36]. Additionally, infectious clones fused with a reporter gene, for instance, the GFP, are widely used for monitoring virus cell-to-cell migration as well as long-distance movement in infected plants. Moreover, such recombined clones can be applied to analyze the dynamic mechanisms of virus infection in hosts [13,37,38].

TBRV infects a wide range of hosts, leading to yield and quality losses. However, knowledge regarding its pathogenicity and host adaptation is rather limited. Understanding molecular biology is nowadays easier with cDNA infectious clones. The first attempt to obtain the TBRV construct was carried out in 2004, when the full length of the cDNA of both RNAs was used in the yeast transformation protocol [26]. Obtained clones were inoculated using a hand-held biolistic device on *C. quinoa* plants, but the constructs were not infectious [26]. The successful constitution was conducted in 2017, when the full length of the cDNA of both RNAs was cloned into a pJL89 binary vector and infiltrated into the plants by agroinoculation. Virus tracking technologies are crucial for studying the virus replication process and understanding the mechanisms involved in the virus life cycle, including virus adsorption and internalization, transportation, genome delivery, assembly, and egress [39]. Therefore, the goal of this study was to develop stable TBRV-based constructs suitable for expressing a reporter protein, GFP, in plants. Choosing an appropriate strategy for engineering the infectious clone is crucial for experiment success; therefore, in the current study, we tested different methods to obtain the most suitable and stable TBRV-GFP clone. This is exceptionally important in the case of virus genera, where the expression of virus proteins undergoes polyprotein maturation. This is the case of pathogens from the *Secoviridae* family, where the TBRV belongs. For TBRV, the putative cleavage sites within polyproteins have been predicted [26,40], but their exact position is still uncertain. Considering that TBRV RNA1 encodes proteins necessary for virus replication, the GFP coding sequence was inserted nowhere within this region. The negative effects of adding a GFP cassette within RNA 1 were described for the lettuce infectious yellow virus (LIYV), where the infectivity of the RNA1 construct was abolished [41]. Therefore, in this work, we decided to insert a GFP coding sequence (flanked by 5 or 20 aa on the N- and C-termini) within predicted cleavage sites on the RNA2 polyprotein. Additionally, we tested the insertion of the GFP coding sequence with a 2A self-cleaving peptide at the C-terminus of the CP ORF, which enabled the release of the GFP from the polyprotein.

The first strategy of engineering the pJL89-TBRV-P1-R2-GFP construct implemented the duplication sequences flanking the GFP coding sequence. The selection of flanking sequences containing the appropriate protein cleavage sites, which are recognized by protease, is extremely important during clone construction. Additionally, duplicated sequences in viral genomes could affect clone stability, cutting protease efficiency, or the risk of being eliminated via homologous recombination. Decreasing protease efficiency in releasing the GFP was shown in a study using the tomato marchitez virus (ToMarV) pJL89-M-R2-GFP clone [14], where GFP was placed between the MP and the VP35 CP. The differences in the length of the GFP flanking sequences of the cleavage site of cowpea mosaic virus (CPMV) impacted vector stability and systemic infection efficiency [42], while in poliovirus-based vectors, a 37% reduction in the homology of the sequences flanking additional genes increased the clone stability [43]. The length of flanking sequences could also affect virus infection, gfp expression, and virus movement ability. In this study, differences in infection efficiency between MP/5/GFP/5/CP and MP/20/GFP/20/CP clones (Table 1) and intensity of developed symptoms were observed; however, no significant divergence in GFP expression under UV light was noticed (Figure 2, Figure 3 and Figure 4). Nevertheless, increasing the number of amino acids to 30 in GFP flanking sequences enabled the clone to move systemically and influence clone infectivity [44]. The infection symptoms of the engineering MP/30/GFP/30/CP construct were observed only on infiltrated leaves of *C. sativus* and *N. tabacum* cv. Xanthi. The slight GFP-derived fluorescence was detected only in infiltrated leaves, mostly covering the inoculation site, and no fluorescence was observed in upper non-inoculated leaves. There are many examples of different lengths of flanking sequences used for the virus-GFP construct [2,5,13,14,42,45], suggesting that this is an individual/virus species-dependent/specific mechanism.

In our next experiment, we decided to change the insertion site of the GFP coding sequence within TBRV RNA2 from MP/CP to HP/MP. In this case, the number of infected plants was slightly lower compared to the first two constructs, but the symptoms of virus infection on infiltrated plants were similar. For all constructs, GFP fluorescence in infiltrated plants was observed.

Another way to develop viral vectors for foreign protein expression is to remove a gene that is not essential for replication and movement and substitute it with the gene of interest. We chose to replace the HP ORF with the GFP coding sequence and obtained the pJL89-P1-R2-HP∆GFP clone [44]. When *A. tumefaciens* harboring HP∆GFP was co-infiltrated with pJL89-P1-R1 into *N. tabacum* cv. Xanthi, no GFP-derived fluorescence was observed in inoculated and upper non-inoculated leaves under UV light. This clone was not able to infect host plants. The role of HP has not been fully studied yet, and those results might suggest that it is essential for virus infection. Nevertheless, this {Citation}strategy was successfully used in the research on LIYV, where the P5 ORF was replaced by the GFP ORF [41]. The engineered LIYV vector was capable of systemic GFP expression in *N. benthamiana* plants, but at a much reduced efficiency compared to other constructs.

The last strategy to produce GFP used in this study was the fusion of pJL89-TBRV-P1-R2 and the GFP cloning sequence with FMDV 2A catalytic peptide. The FMDV 2A has a covalently linked autocleavage site with a unique ribosome skipping property [46]. Introducing the sequence encoding FMDV 2A allowed the direct, highly effective release of GFP from a TBRV RNA2 polyprotein. The constructed clone showed the highest infectivity and expressed more visible symptoms compared to other constructs (Table 1). The fusion protein 2A-GFP was used to develop an expression vector based on pepino mosaic virus (PepMV) and gave the best results of maintaining stability through serial passages and GFP accumulation in comparison to coat protein (CP) replacement or duplication of the CP subgenomic promoter (SGP) [47]. The same results were obtained for CPMV, where insertion of the FMDV 2A sequence at the C-terminus of the GFP resulted in a genetically stable construct of engineered CPMV [42] or for pepper ringspot virus (PepRSV), where the CP gene, 2A self-cleavage peptide, and mGFP5 gene were fused [48].

By using RT-PCR, we confirmed that all constructs were able to systemically infect the four tested host plants. Symptoms observed on agroinfiltrated plants were identical to those observed on plants infected with the wild-type (wt) TBRV-P1 isolate. For both TBRV-GFP and TBRV-wt, chlorotic lesions on leaves of *C. sativus*, *C. quinoa*, and *N. tabacum* cv. Xanthi were observed, as well as mosaics and systemic ringspots on *N. tabacum* cv. Xanthi. Infection in *C. quinoa* plants developed earlier, leading to more severe symptoms and plant death, while symptoms in *C. sativus* were noticed relatively later in comparison to other host plants. *N. benthamiana* plants remain symptomless. Interestingly, *N. tabacum* cv. Xanthi, *C. quinoa*, and *C. sativus* plants inoculated with the sap from infected *N. benthamiana* exhibited clear TBRV symptoms and expressed significant GFP fluorescence, while mechanically inoculated *N. benthamiana* plants remained symptomless. Taking together, all the constructs were able to back-infect new sets of host plants showing the same symptoms as plants agroinfiltrated with infectious clones (Supplementary File S2). 

## 5. Conclusions

In this work, we generated four cDNA infectious clones of TBRV-GFP. All the analyses performed confirmed that heterologous GFP can be produced in plants using the mentioned infectious TBRV-GFP clones. The obtained cDNA clones (especially the CP/2A/GFP) provide a valuable research tool to work on gene function and pathogenesis of the virus and to determine important biological properties such as interactions between TBRV and its host plants or adaptation to multiple hosts. This knowledge is essential to establishing the most appropriate and economical approach to crop protection.

## Figures and Tables

**Table 1:** The presence of both pJL89-TBRV-P1-R1 and pJL89-TBRV-P1-R2-GFP in host plants agroinfiltrated with different TBRV-GFP constructs was confirmed by RT-PCR.

Host/Clone	MP/5/GFP/5/CP	MP/20/GFP/20/CP	HP/20/GFP/20/MP	CP/2A/GFP
*N. benthamiana*	1/4	4/4	3/4	4/4
*N. tabacum* cv. Xanthi	2/4	3/4	2/4	2/4
*C. sativus*	3/4	4/4	2/4	4/4
*C. quinoa*	3/4	3/4	2/4	4/4

## Data Availability

Data are contained within the article and Supplementary Materials.

## Supplementary Materials

The following supporting information can be downloaded at: https://www.mdpi.com/article/10.3390/v16010125/s1. Supplementary File S1. Schematic representation of the amplifying TBRV RNA2 genome fragments containing gfp insertion used for each constructed clone. Details regarding the used primer pairs are implemented in Supplementary Table S2. Supplementary File S2. Results of the mechanical inoculation experiment confirm the construct’s stability and infectibility. Supplementary Table S1. Primers were used to obtain the GFP-tagged infectious TBRV clones. Supplementary Table S2. Primers were used to perform colony PCR and detection of GFP insertion as well as TBRV-pJL89-P1-R1. Supplementary Table S3. Oligonucleotides are used to obtain the 2A self-cleaving peptide of the foot-and-mouth disease virus (FMDV).

## References

[1] Ahlquist P., Dasgupta R., Kaesberg P. (1984). Nucleotide Sequence of the Brome Mosaic Virus Genome and Its Implications for Viral Replication. J. Mol. Biol..

[2] Li X., Li Y., Chen S., Wang J. (2020). Construction of Stable Infectious Full-Length and eGFP-Tagged cDNA Clones of Mirabilis Crinkle Mosaic Virus via In-Fusion Cloning. Virus Res..

[3] Cheng D.-J., Tian Y.-P., Geng C., Guo Y., Jia M.-A., Li X.-D. (2020). Development and Application of a Full-Length Infectious Clone of Potato Virus Y Isolate Belonging to SYR-I Strain. Virus Res..

[4] Masuta C., Yamana T., Tacahashi Y., Uyeda I., Sato M., Ueda S., Matsumura T. (2000). Development of Clover Yellow Vein Virus as an Efficient, Stable Gene-Expression System for Legume Species. Plant J..

[5] Yin J., Hong X., Luo S., Tan J., Zhang Y., Qiu Y., Latif M.F., Gao T., Yu H., Bai J. (2022). The Characterization of the Tobacco-Derived Wild Tomato Mosaic Virus by Employing Its Infectious DNA Clone. Biology.

[6] Zheng H., Xiao C., Han K., Peng J., Lin L., Lu Y., Xie L., Wu X., Xu P., Li G. (2015). Development of an Agroinoculation System for Full-Length and GFP-Tagged cDNA Clones of Cucumber Green Mottle Mosaic Virus. Arch. Virol..

[7] Casper S.J., Holt C.A. (1996). Expression of the Green Fluorescent Protein-Encoding Gene from a Tobacco Mosaic Virus-Based Vector. Gene.

[8] Lee M.Y., Song Y.S., Ryu K.H. (2011). Development of Infectious Transcripts from Full-Length and GFP-Tagged cDNA Clones of Pepper Mottle Virus and Stable Systemic Expression of GFP in Tobacco and Pepper. Virus Res..

[9] MacFarlane S.A., Popovich A.H. (2000). Efficient Expression of Foreign Proteins in Roots from Tobravirus Vectors. Virology.

[10] Sadowy E., Pluta K., Gronenborn B., Hulanicka D. (1998). Infectious Transcripts from Cloned cDNA of Potato Leafroll Luteovirus. Acta Biochim. Pol..

[11] Franco-Lara L.F., McGeachy K.D., Commandeur U., Martin R.R., Mayo M.A., Barker H. (1999). Transformation of Tobacco and Potato with cDNA Encoding the Full-Length Genome of Potato Leafroll Virus: Evidence for a Novel Virus Distribution and Host Effects on Virus Multiplication. J. Gen. Virol..

[12] Leiser R.M., Ziegler-Graff V., Reutenauer A., Herrbach E., Lemaire O., Guilley H., Richards K., Jonard G. (1992). Agroinfection as an Alternative to Insects for Infecting Plants with Beet Western Yellows Luteovirus. Proc. Natl. Acad. Sci. USA.

[13] Wieczorek P., Budziszewska M., Frąckowiak P., Obrępalska-Stęplowska A. (2020). Development of a New Tomato Torrado Virus-Based Vector Tagged with GFP for Monitoring Virus Movement in Plants. Viruses.

[14] Ferriol I., Turina M., Zamora-Macorra E.J., Falk B.W. (2016). RNA1-Independent Replication and GFP Expression from Tomato Marchitez Virus Isolate M Cloned cDNA. Phytopathology.

[15] Zarzyńska-Nowak A., Ferriol I., Falk B.W., Borodynko-Filas N., Hasiów-Jaroszewska B. (2017). Construction of Agrobacterium Tumefaciens-Mediated Tomato Black Ring Virus Infectious cDNA Clones. Virus Res..

[16] Kumar M., Zarreen F., Chakraborty S. (2021). Roles of Two Distinct Alphasatellites Modulating Geminivirus Pathogenesis. Virol. J..

[17] Stevens M., Viganó F. (2007). Production of a Full-Length Infectious GFP-Tagged cDNA Clone of Beet Mild Yellowing Virus for the Study of Plant–Polerovirus Interactions. Virus Genes.

[18] Hasiów-Jaroszewska B., Zarzyńska-Nowak A. (2022). Tomato Black Ring Virus (Ring Spot of Beet). CABI Compend..

[19] Pospieszny H., Borodynko-Filas N., Hasiów-Jaroszewska B., Czerwonka B., Elena S.F. (2019). An Assessment of the Transmission Rate of Tomato Black Ring Virus through Tomato Seeds. Plant Prot. Sci..

[20] Tomato Black Ring Virus (TBRV00) [World Distribution]|EPPO Global Database. https://gd.eppo.int/taxon/TBRV00/distribution.

[21] Dewasme C., Mary S., Darrieutort G., Audeguin L., van Helden M., van Leeuwen C. (2019). Impact of Tomato Black Ring Virus (TBRV) on Quantitative and Qualitative Features of *Vitis vinifera* L. Cv. Merlot and Cabernet Franc. OENO One.

[22] Gallitelli D., Rana G.L., Vovlas C., Martelli G.P. (2004). Viruses of Globe Artichoke: An Overview. J. Plant Pathol..

[23] Dąbrowska E., Paduch-Cichal E., Piasna P., Malewski T., Mirzwa-Mróz E. (2021). First Report of Tomato Black Ring Virus Infecting Raspberry and Blackberry in Poland. Plant Dis..

[24] Al-Shahwan I., Al-Shudifat A., Al-Saleh M., Abdalla O., Amer M. (2021). First Report of Tomato Black Ring Virus on Tomato (*Solanum lycopersicum*) in Saudi Arabia. Plant Dis..

[25] Oncino C., Hemmer O., Fritsch C. (1995). Specificity in the Association of Tomato Black Ring Virus Satellite RNA with Helper Virus. Virology.

[26] Jończyk M., Le Gall O., Pałucha A., Borodynko N., Pospieszny H. (2004). Cloning and Sequencing of Full-Length cDNAs of RNA1 and RNA2 of a Tomato Black Ring Virus Isolate from Poland. Arch. Virol..

[27] Hasiów-Jaroszewska B., Minicka J., Zarzyńska-Nowak A., Budzyńska D., Elena S.F. (2018). Defective RNA Particles Derived from Tomato Black Ring Virus Genome Interfere with the Replication of Parental Virus. Virus Res..

[28] Gnanasekaran P., Chakraborty S. (2018). Biology of Viral Satellites and Their Role in Pathogenesis. Curr. Opin. Virol..

[29] Hasiów-Jaroszewska B., Borodynko N., Figlerowicz M., Pospieszny H. (2012). Two Types of Defective RNAs Arising from the Tomato Black Ring Virus Genome. Arch. Virol..

[30] Budzyńska D., Minicka J., Hasiów-Jaroszewska B., Elena S.F. (2020). Molecular Evolution of Tomato Black Ring Virus and de Novo Generation of a New Type of Defective RNAs during Long-Term Passaging in Different Hosts. Plant Pathol..

[31] Minicka J., Taberska A., Zarzyńska-Nowak A., Kubska K., Budzyńska D., Elena S.F., Hasiów-Jaroszewska B. (2022). Genetic Diversity of Tomato Black Ring Virus Satellite RNAs and Their Impact on Virus Replication. Int. J. Mol. Sci..

[32] Pelczyk M., Obrępalska-Stęplowska A., Pospieszny H. (2006). Subviral Molecules of RNA Associated with Plant Ss(+)RNA Viruses. Postep. Biochem..

[33] Hemmer O., Oncino C., Fritsch C. (1993). Efficient Replication of the in Vitro Transcripts from Cloned cDNA of Tomato Black Ring Virus Satellite RNA Requires the 48K Satellite RNA-Encoded Protein. Virology.

[34] Zarzyńska-Nowak A., Hasiów-Jaroszewska B., Budzyńska D., Trzmiel K. (2020). Genetic Variability of Polish Tomato Black Ring Virus Isolates and Their Satellite RNAs. Plant Pathol..

[35] Zarzyńska-Nowak A., Jeżewska M., Hasiów-Jaroszewska B., Zielińska L. (2015). A Comparison of Ultrastructural Changes of Barley Cells Infected with Mild and Aggressive Isolates of Barley Stripe Mosaic Virus. J. Plant Dis. Prot..

[36] Brewer H.C., Hird D.L., Bailey A.M., Seal S.E., Foster G.D. (2018). A Guide to the Contained Use of Plant Virus Infectious Clones. Plant Biotechnol. J..

[37] Oparka K.J., Boevink P., Santa Cruz S. (1996). Studying the Movement of Plant Viruses Using Green Fluorescent Protein. Trends Plant Sci..

[38] Zhou X., Lin W., Sun K., Wang S., Zhou X., Jackson A.O., Li Z. (2019). Specificity of Plant Rhabdovirus Cell-to-Cell Movement. J. Virol..

[39] Liu D., Pan L., Zhai H., Qiu H.-J., Sun Y. (2023). Virus Tracking Technologies and Their Applications in Viral Life Cycle: Research Advances and Future Perspectives. Front. Immunol..

[40] Digiaro M., Yahyaoui E., Martelli G.P., Elbeaino T. (2015). The Sequencing of the Complete Genome of a Tomato Black Ring Virus (TBRV) and of the RNA2 of Three Grapevine Chrome Mosaic Virus (GCMV) Isolates from Grapevine Reveals the Possible Recombinant Origin of GCMV. Virus Genes.

[41] Qiao W., Falk B.W. (2018). Efficient Protein Expression and Virus-Induced Gene Silencing in Plants Using a Crinivirus-Derived Vector. Viruses.

[42] Gopinath K., Wellink J., Porta C., Taylor K.M., Lomonossoff G.P., Van Kammen A. (2000). Engineering Cowpea Mosaic Virus RNA-2 into a Vector to Express Heterologous Proteins in Plants. Virology.

[43] Tang S., van Rij R., Silvera D., Andino R. (1997). Toward a Poliovirus-Based Simian Immunodeficiency Virus Vaccine: Correlation between Genetic Stability and Immunogenicity. J. Virol..

[44] Zarzyńska-Nowak A., Minicka J., Wieczorek P., Hasiów-Jaroszewska B. Infekcyjne kopie TBRV sprzężone z białkiem zielonej fluorescencji jako narzędzie do analizy przebiegu patogenezy w tkankach roślinnych. Proceedings of the 62 Sesja Naukowa Instytutu Ochrony Roślin—Państwowego Instytutu Badawczego.

[45] Zhang C., Ghabrial S.A. (2006). Development of Bean Pod Mottle Virus-Based Vectors for Stable Protein Expression and Sequence-Specific Virus-Induced Gene Silencing in Soybean. Virology.

[46] Zhang B., Rapolu M., Kumar S., Gupta M., Liang Z., Han Z., Williams P., Su W.W. (2017). Coordinated Protein Co-Expression in Plants by Harnessing the Synergy between an Intein and a Viral 2A Peptide. Plant Biotechnol. J..

[47] Sempere R.N., Gómez P., Truniger V., Aranda M.A. (2011). Development of Expression Vectors Based on Pepino Mosaic Virus. Plant Methods.

[48] Tavares-Esashika M.L., Campos R.N.S., Maeda M.H.K., Koyama L.H.H., Hamann P.R.V., Noronha E.F., Nagata T. (2022). Development of a Heterologous Gene Expression Vector in Plants Based on an Infectious Clone of a Tobravirus, Pepper Ringspot Virus. Ann. Appl. Biol..

