# Supplementary material for: Development of Stable Infectious cDNA Clones of Tomato Black Ring Virus Tagged with Green Fluorescent Protein
Source: Viruses. 2024 Jan 15;16(1):125. doi: 10.3390/v16010125 (PMC10819210; doi:10.3390/v16010125)
Supplement: Supplementary file 1 [file viruses-16-00125-s001.zip › Supplementary Table S1.pdf]

Supplementary Table S1
Primers used to obtain the GFP-tagged infectious TBRV clones.

GFP-TBRV Clone	Objective	Name	Sequence (5'->3')
MP/5/GFP/5/CP	vector	G5 GFP vector F	GCTGATGGAGACTTCGCCTGTGGTGAAACCATCACC
		G6 GFP vector R	TTTTAAGTTGCAGCTTGATTCATCAAGACGGGGTTGATT
	insert	G7 GFP insert F	GAATCAAGCTGCAACTTAAAAGCGGATGGAGACTTCATGGTGAGCAAGGGCGAG
		G4 GFP insert R	GTTTCACCACAGGCGAAGTCTCCATCAGCTTTTAAGTTGCAGCTCTTGTACAGCTCG
MP/20/GFP/20/CP	vector	pJL_RNA2_casPW_F	TAACCAGCCGCGACTGGACGAGAGCTCCTGCAACCTCAAAGCAGACGGGGATTTTGCCT
		pJL_RNA2_casPW_R	GCAATGTAATCGTCTCGCCGCACGCGAAGTCGCCATCCGCCTTCAAATTACAACTTGATTCATCAAGAC
	insert	sGFP_PW_F	CGGCGAGACGATTACATTGCCGGCGACCTCCGCCTCCGGCATGGTGAGCAAGGGCGAG
		sGFP_PW_R	CGTCCAGTCGCGGCTGGTTAGAGGTGCTCGCTCTGGCCAACTTGTACAGCTCGTCCATGC
HP/20/GFP/20/MP	vector	P1 HP-GFP-MP vector F	GACTGCGCTGTCTGTTTCCCAGGCTCCGTATATAAGAGGCTGAATGGAGGAG
		P1 HP-GFP-MP vector R	TCGGCACCAATCATCGTAACCTTTACCGCCACCGTTGAGTCTTTTGTAGACCGAACCAGGAAAGC
	insert	P1 HP-GFP-MP insert F	GGTTACGATGATTGGTGCCGACTACCGCCCAATAAAGAGATGGTGAGCAAGGGCGAG
		P1 HP-GFP-MP insert R	TGGGAAACAGACAGCGCAGTCCTCCTCGTCGAGTAGGATCTGAGCCTTGTACAGCTCGTCCATG
pJL89-P1-R2 + 2A	vector	2A Vector.FOR	ATTCTTAGGCATTTCTTATAGAGAATATCCCTCCC
		2A Vector.REV	TGCAGGAATAGTTAAAGGACCTGCACTTC
	insert	2A Fragment F2	TTAACTATTCCTGCAGGAAGCGGAGTGAAACAGACTT
		2A Fragment R2	GAAATGCCTAAGAATAGGTCCAGGGTTGGACTCCACGTC

CP/2A/GFP	vector	2A Vector F	ATTCTTAGGCATTTCTTATAGAGAATATCCCTCCC
		2AGFP Vector R	TGCAGGAATAGTTAAAGGACCTGCACTTC
	insert	2AGFP Fragment 2F	CCCTGGACCTATGGTGAGCAAG
		2AGFP Fragment 2R	TATAAGAAATGCCTAAGAATCTTGTACAGCTCGT
