# Supplementary material for: Development of Stable Infectious cDNA Clones of Tomato Black Ring Virus Tagged with Green Fluorescent Protein
Source: Viruses. 2024 Jan 15;16(1):125. doi: 10.3390/v16010125 (PMC10819210; doi:10.3390/v16010125)
Supplement: Supplementary file 1 [file viruses-16-00125-s001.zip › Supplementary Table S2.pdf]

Supplementary Table S2

Primers used to perform colony PCR and detection GFP insertion as well as TBRV-pJL89-P1-R1.

GFP-TBRV clone	Name	Sequence (5'->3')
MP/5/GFP/5CP<br>MP/20/GFP/20/CP	seqMPCPF	CTGGCAACATTCCTGTTGGT
	seqMPCPR	AACACGTGTGTTCGTATTCTTGA
HP/20/GFP/20/MP	R2MJ2F	CTGATGAAGACCTATGTGATGT
	R2MJ2R	CATCCCGCTTCAGCAAGTTTA
pJL89-P1-R2 + 2A<br>CP/2A/GFP	3RACER2PR1	TGACTGTTAGCATAGAGGTTCA
	pJL89seqR	GGGAAATTCGAGCTCTCCCT
TBRV-pJL89-P1-R1	R1KRTBRVF	GGTAAAAGTTCTGGGTGCT
	R1KRTBRVR	GCAAATCCACCTCCTTATCC
