# Supplementary material for: Development of Stable Infectious cDNA Clones of Tomato Black Ring Virus Tagged with Green Fluorescent Protein
Source: Viruses. 2024 Jan 15;16(1):125. doi: 10.3390/v16010125 (PMC10819210; doi:10.3390/v16010125)
Supplement: Supplementary file 1 [file viruses-16-00125-s001.zip › Supplementary Table S3.pdf]

Supplementary Table S3

Oligonucleotides used to obtain 2A self-cleaving peptide of the foot-and-mouth disease virus (FMDV).

**Name**	**Sequence (5’-> 3’)**
2AFMDV_F	AAGGAAGCGGAGTGAAACAGACTTTGAATTTTGACCTTCT CAAGTTGGCGGGAGACGTGGAGTCCAACCCTGGACCTAA
2AFMDV_R	AAAGGTCCAGGGTTGGACTCCACGTCTCCCGCCAACTTGA GAAGGTCAAAATTCAAAGTCTGTTTCACTCCGCTTCCAA
