# Supplementary figures and images for: Development of Stable Infectious cDNA Clones of Tomato Black Ring Virus Tagged with Green Fluorescent Protein
Source: Viruses. 2024 Jan 15;16(1):125. doi: 10.3390/v16010125 (PMC10819210; doi:10.3390/v16010125)

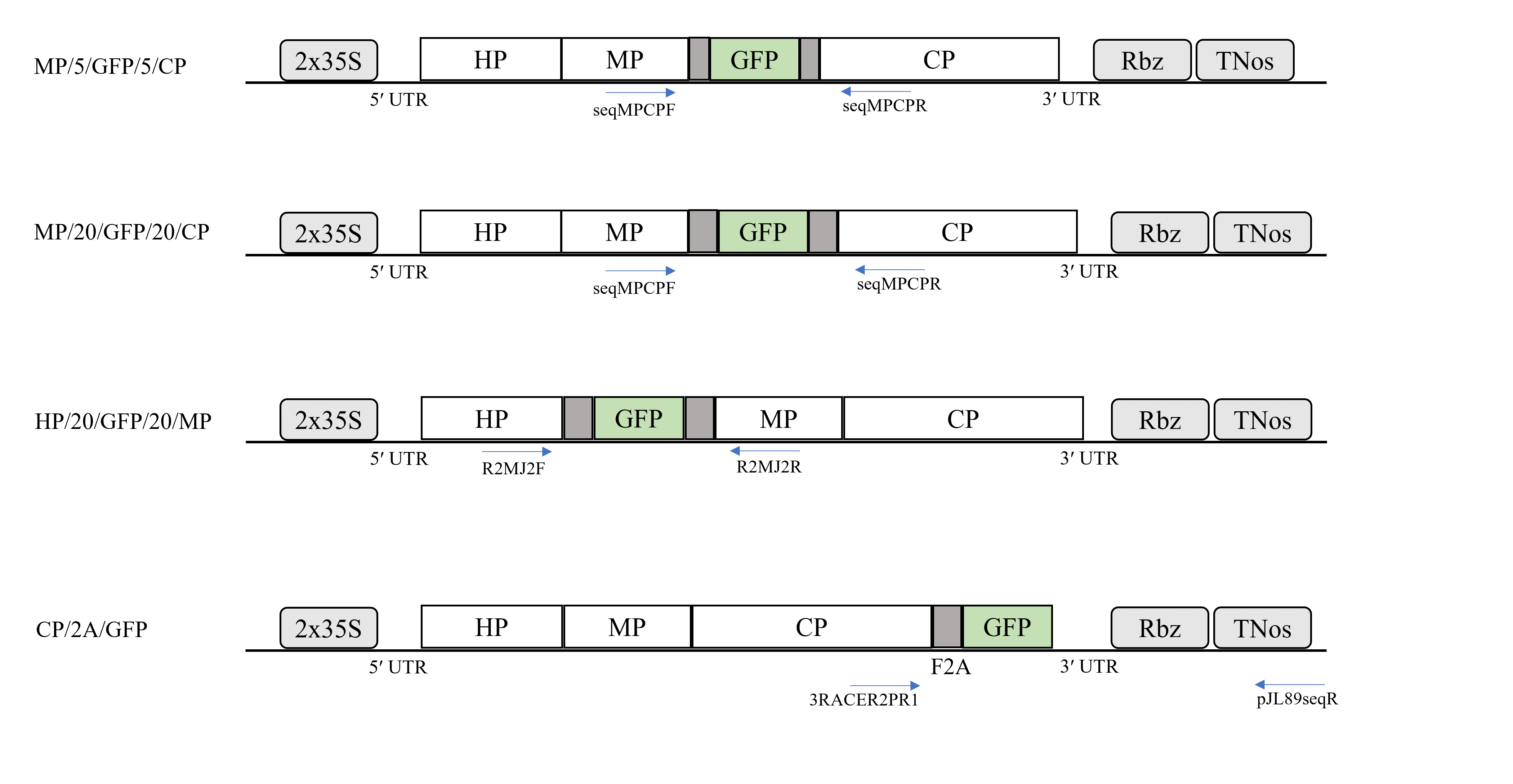

Supplement: Supplementary file 1 [file viruses-16-00125-s001.zip › Supplementary file 1.png]

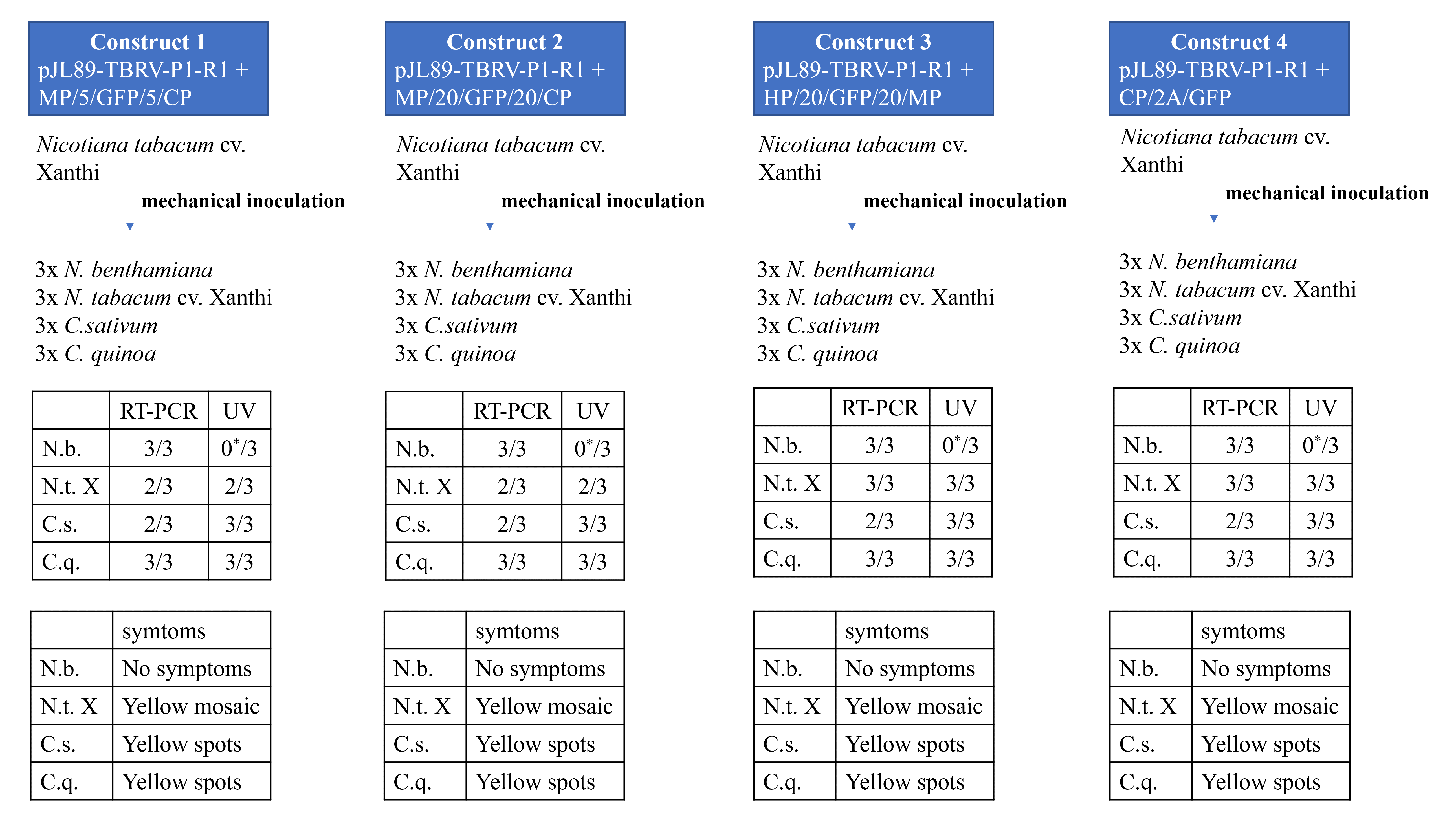

Supplement: Supplementary file 1 [file viruses-16-00125-s001.zip › Supplementary file 2.png]
